# Interview Versus Performance Assessment of Cognition as Predictors of Real-World Outcomes in a Large-Scale Cross-Sectional Study in Schizophrenia

**DOI:** 10.1093/schizbullopen/sgae020

**Published:** 2024-08-12

**Authors:** Pasquale Pezzella, Edoardo Caporusso, Armida Mucci, Paola Bucci, Giulia M Giordano, Mario Amore, Paola Rocca, Alessandro Rossi, Alessandro Bertolino, Joseph Ventura, Silvana Galderisi, Mario Maj, Italian Network for Research on Psychoses, Luigi Giuliani, Andrea Perrottelli, Giuseppe Piegari, Eleonora Merlotti, Daria Pietrafesa, Francesco Brando, Noemi Sansone, Antonio Melillo, Marco Papalino, Vitalba Calia, Raffaella Romano, Pietro Calcagno, Martino Belvedere Murri, Simone Cattedra, Cristiana Montemagni, Cecilia Riccardi, Elisa Del Favero, Francesca Pacitti, Rodolfo Rossi, Valentina Socci

**Affiliations:** Department of Mental and Physical Health and Preventive Medicine, University of Campania “Luigi Vanvitelli,” Naples, Italy; Department of Mental and Physical Health and Preventive Medicine, University of Campania “Luigi Vanvitelli,” Naples, Italy; Department of Mental and Physical Health and Preventive Medicine, University of Campania “Luigi Vanvitelli,” Naples, Italy; Department of Mental and Physical Health and Preventive Medicine, University of Campania “Luigi Vanvitelli,” Naples, Italy; Department of Mental and Physical Health and Preventive Medicine, University of Campania “Luigi Vanvitelli,” Naples, Italy; Department of Neurosciences, Rehabilitation, Ophthalmology, Genetics and Maternal and Child Health, Section of Psychiatry, University of Genoa, Genoa, Italy; Department of Neuroscience, Section of Psychiatry, University of Turin, Turin, Italy; Department of Biotechnological and Applied Clinical Sciences, Section of Psychiatry, University of L’Aquila, L’Aquila, Italy; Department of Basic Medical Science, Neuroscience and Sense Organs, University of Bari “Aldo Moro,” Bari, Italy; Department of Psychiatry and Biobehavioral Sciences, Semel Institute for Neuroscience and Human Behavior, University of California, Los Angeles, CA, USA; Department of Mental and Physical Health and Preventive Medicine, University of Campania “Luigi Vanvitelli,” Naples, Italy; Department of Mental and Physical Health and Preventive Medicine, University of Campania “Luigi Vanvitelli,” Naples, Italy; University of Campania “Luigi Vanvitelli,” Naples; University of Bari; University of Genoa; University of Turin; University of L’Aquila

**Keywords:** cognitive assessment interview, schizophrenia, cognitive impairment, negative symptoms, social cognition, coprimary measure, functional capacity, real-life functioning

## Abstract

The Cognitive Assessment Interview (CAI) is an interview-based scale measuring cognitive impairment and its impact on functioning in subjects with schizophrenia (SCZ). It is approved as a coprimary measure of performance-based instruments, such as the Measurement and Treatment Research to Improve Cognition in Schizophrenia Consensus Cognitive Battery (MCCB). Recent research highlights negative symptoms, social cognition, and functional capacity as mediators of cognitive impairment’s impact on functioning. This study compared mediation analysis outcomes using CAI or MCCB scores, providing insights into the utility of interview-based tools in research and clinical practice. The study included 618 individuals diagnosed with schizophrenia, recruited from 24 Italian psychiatric clinics. Neurocognitive assessments utilized both CAI and MCCB. Mediation analyses explored negative symptoms, social cognition, and functional capacity as mediators of the impact of neurocognition on real-life functioning domains. The study’s results extend the validation of the CAI as a coprimary measure that provides valid information on the impact of cognitive impairment on real-life functioning and its possible mediators, complementing the information obtained using the MCCB. Interview-based cognitive assessment might be essential for understanding schizophrenia complexity and its impact on various cognitive and functional domains for clinicians, patients, and caregivers.

## Introduction

The impairment in different cognitive domains, such as attention, memory, verbal learning, visual learning, reasoning/problem-solving, and processing speed^1,2^ has been widely reported in schizophrenia. It is considered a core feature of the disorder.^3–5^ These deficits can manifest before the onset of the disorder,^6,7^ often persist even after symptom remission and during periods of clinical stability,^8^ and have a direct or indirect association (mediated by other variables) with several aspects of functioning, ie, work skills and everyday life skills.^9–14^ In addition, they have been identified, albeit to a lesser extent, in unaffected first-degree relatives of individuals with schizophrenia,^8^ suggesting a potential vulnerability factor for the disorder. Extensive literature findings consistently indicate that cognitive deficits rank among the most robust predictors of functional outcomes in individuals with schizophrenia, exerting a more pronounced influence on everyday life functioning than positive and negative symptoms.^9–11,15–18^

To evaluate cognitive deficits comprehensively, a panel of experts developed the NIMH-Measurement and Treatment Research to Improve Cognition in Schizophrenia (MATRICS) Consensus Cognitive Battery (MCCB),^19,20^ a performance-based instrument that has become recognized as the gold standard for detecting cognitive impairment in individuals with schizophrenia.

However, beyond these standardized, performance-based tools, the US Food and Drug Administration (FDA), as part of the MATRICS initiative, highlighted the need to integrate primary measures of cognitive functioning with coprimary measures, including interview-based assessment instruments.^21^

Both the performance-based and the interview-based evaluation tools have been developed to assess how cognitive impairment influences functioning, offering potential utility in both clinical trials and routine practice, aiding patients and clinicians in evaluating the clinical significance of cognitive impairment, as well as its progression over time or in response to pharmacological or psychosocial interventions.^22–25^

Standardized neuropsychological assessment is considered the gold standard in research on cognitive impairment; however, it should be noted that interview-based cognitive assessments offer several advantages: (1) they are more time-convenient, practical and easier to use in clinical settings compared to extensive neuropsychological test batteries; (2) they enable patients and caregivers to have a better insight of the presence of cognitive deficits, which may increase motivation to adhere to cognitive rehabilitation programs and promote awareness of the impact of cognitive deficits on real-life functioning; (3) they can uncover subtle cognitive deficits even when performance on neuropsychological tests falls within the normal range; (4) they may enable the detection of cognitive improvements induced by treatments that are only subjectively perceived; and (5) they facilitate the awareness of how cognition affects daily functioning in a manner that is understandable not only for healthcare professionals, but also for patients and caregivers; on the other hand, neuropsychological test scores do not directly provide information regarding how patients’ cognitive deficits influence their everyday functioning, and their longitudinal changes may have no immediate meaning for patients and caregivers.^22,26–29^

The MATRICS Initiative examined interview-based cognitive instruments as coprimary measures of cognition in schizophrenia. Among those, the Schizophrenia Cognition Rating Scale (SCoRS)^27^ and the Clinical Global Impression of Cognition in Schizophrenia (CGI-CogS)^29^ showed good psychometric properties. They were recognized as valid coprimary measures in line with the MATRICS Initiative’s intentions, being linked to the composite score of MCCB and measures of functional capacity and functioning.^22,23,27^ In addition, SCoRS has been widely used in many different clinical trials^30^ and is sensitive to pharmacological treatment effects in a randomized blinded trial.^31^ However, these instruments showed some limitations. Specifically, the relationship between scores on SCoRS and functioning was observed only in clinically stable patients, not in recently hospitalized ones, indicating the limited utility of SCoRS in acute phases.^32^ Additionally, limitations of these coprimary measures included their tendency to assess overall cognitive impairment without focusing on specific cognitive deficits.^28^ Moreover, item-response theory analysis revealed that fewer items from the SCoRS and CGI-CogS scales were enough to accurately estimate neuropsychological deficits.^28^

The Cognitive Assessment Interview (CAI)^33^ was developed to overcome these issues. The CAI is an interview-based tool that shortens and modifies the CGI-Cogs and the SCoRS scales. It has been developed for assessing cognition and is designed to consider how cognitive impairment affects functioning. The CAI exhibited good to excellent psychometric properties, including reliability, internal consistency, and a manageable administration time of 15–30 minutes.^26^ Importantly, it did not show practice effects; thus, it could be reliably used to detect changes over time.^33–37^ Moreover, it was found to correlate with measures of neurocognition, functional capacity, and everyday functioning.^33,34,36–39^ CAI scores were found to better reflect the influence of cognitive impairment on everyday functioning in individuals with schizophrenia compared to objective measures.^33,34,36–38,40^

The CAI and other interview-based assessment instruments have been validated and utilized as a coprimary measure. However, associations between CAI scores and real-life functioning and between CAI and MCCB scores have not always been found.^21,33,34,36–40^

Understanding how the interview-based assessments align with performance-based measures might shed light on their complementary roles in evaluating cognitive impairment in schizophrenia.

Recent research has highlighted the importance of considering moderators and mediators of the impact of cognitive impairment on functioning.^9,10,15,41–44^ For instance, recent findings highlighted the role of negative symptoms in mediating the effects of cognitive impairment on functioning^41,42^ and in moderating the functional gain associated with cognitive remediation.^44^ Well-known mediators of the impact of neurocognition on functional outcomes are social cognition and functional capacity.^9,10^ Comparing the mediation analysis results on the impact of neurocognition on functioning when using the MCCB or the CAI to assess cognitive deficits could provide further insights into the potential utility of interview-based tools in research and clinical practice.

In this regard, the present study aims to compare, for the first time, the results of mediation analyses by assessing neurocognition using either CAI or MCCB scores.

Carried out in a large sample of stabilized individuals diagnosed with schizophrenia as part of a national multicenter study, this study aimed to further extend the validation of the CAI by demonstrating that it provides information on cognitive impairment comparable with that obtained by using the MCCB and can be used to validly investigate whether other variables partially or fully mediate the effects of cognitive impairment on functional outcomes. Therefore, we sought to assess the roles of negative symptoms, social cognition, and functional capacity in mediating the impact of neurocognitive deficits on real-life functioning among individuals with schizophrenia and to compare results obtained using the CAI and the MCCB in the mediation analysis.

## Methods

The present study was carried out in a large sample of community-dwelling persons with schizophrenia within the activities of the Italian Network for Research on Psychoses (NIRP). We used the database relevant to the 4-year follow-up study^9,16^ since the CAI interview was not included in the baseline assessments.

The study participants were patients recruited among those consecutively seen at the outpatient units of 24 Italian university psychiatric clinics and/or mental health departments. Inclusion criteria were a diagnosis of schizophrenia confirmed with the Structured Clinical Interview for DSM-IV-Patient version (SCID-I-P) and an age between 18 and 66 years. Exclusion criteria were: (1) history of head trauma with loss of consciousness in the last 4 years; (2) progressive cognitive deterioration possibly due to dementia or other neurological illness diagnosed in the last 4 years; (3) history of alcohol and/or substance abuse in the last 6 months; (4) current pregnancy or lactation; (5) inability to provide informed consent; and (6) treatment modifications (any change in the antipsychotic treatment, either dosage or compound) and/or hospitalization due to symptom exacerbation in the last 3 months to ensure clinical stability of the sample. After receiving a comprehensive explanation of the study procedures and aims, all subjects signed a written informed consent to participate.

The study protocol was approved by the Ethics Committee and has been conducted in accordance with the principles of the Declaration of Helsinki (59th World Medical Association General Assembly; October 2008).

### Assessment

#### Psychopathology.

The Positive and Negative Syndrome Scale (PANSS)^45^ was used to rate the severity of 2 psychopathological dimensions: “Positive symptoms” was calculated according to Wallwork et al.^46^ by summing the scores for “delusions” (P1), “hallucinatory behavior” (P3), “grandiosity” (P5), and “unusual thought content” (G9); “Disorganization”–was represented by the PANSS item “conceptual disorganization” (P2), to avoid overlap with cognitive impairment as the PANSS disorganization factor includes “difficulties in abstract thinking” (N5) and “poor attention” (G11).

Negative symptoms were assessed using the Brief Negative Symptom Scale (BNSS),^47^ an instrument designed to overcome the limitations of the PANSS and other instruments in assessing these symptoms.^48^ The BNSS allows the identification of 2 separate factors: the “Experiential domain,” consisting of anhedonia, asociality, and avolition, and the “Expressive deficit domain” including blunted affect and alogia.^43,49–54^

Depressive symptoms were assessed by means of the Calgary Depression Scale for Schizophrenia (CDSS).^55^ The CDSS includes 9 items (depression, hopelessness, self-depreciation, guilty ideas of reference, pathological guilt, morning depression, early wakening, suicide, observed depression), each rated from 0 (absent) to 3 (severe).

#### Neurocognition—Performance-Based Assessment.

The MATRICS Consensus Cognitive Battery (MCCB)^20,56^ was used for the performance-based neurocognitive assessment. The MCCB includes tests assessing 7 distinct cognitive domains: processing speed, attention/vigilance, working memory, verbal learning, visual learning, reasoning/problem-solving, and social cognition. The latter domain was not used since a thorough assessment of social cognition was included in this study, as described below. Standardized T-scores corrected for age and gender using Italian normative data^8^ were calculated using the same measurement scale with a mean of 50 and SD of 10. The MCCB provides 2 composite score options: the overall composite score and the neurocognitive composite scores, which respectively include and exclude the social cognition domain. We used the latter as an index of neurocognition.

#### Neurocognition—Interview-Based Assessment.

The CAI^33^ is a semi-structured interview developed from a large data set that included the CGI-Cogs^27^ and the SCoRS^57^ scales in which the application of Item Response Theory yielded 10 Items. These ten items assess 6 of the 7 cognitive domains derived from the MCCB (speed of processing, attention/vigilance, working memory, verbal learning and memory, reasoning and problem-solving, and social cognition). Each item is scored from 1 to 7, with higher scores indicating greater impairment. A “not applicable” score is assigned if the subject interrupts the interview or does not provide enough information. The clinician assigns a score rating based on the extent to which cognitive impairment influences expected levels of functioning in the workplace, school, or during social interactions such as with family or friends while minimizing rating the influence on functioning of symptoms of the disorder such as depression or negative symptoms. The CAI interview should be administered to the patient (patient interview) and an informant (informant interview), for instance, a caregiver or someone who knows the patient well enough to comment on how cognitive functioning influences daily functioning. Separate scores are obtained from the patient and the informant interviews. The patient’s interview scores reflect the expert judgment of the clinician exclusively based on the patient’s interview. In contrast, the informant’s interview scores reflect the expert judgment of the clinician based on the informant’s interview. In addition, the clinician assigns for each CAI item a rater composite score, reflecting his/her expert judgment combining that obtained by both interviews (patient and informant) and, when available or applicable, additional sources, eg, chart or other sources of valid information. At the completion of the CAI interview, a score of 1 to 7 is rated on the global severity of cognitive impairment, reflecting the patient’s overall cognitive impairment. Also, for the global score, there are 3 separate ratings (one based on the patient interview, one on the informant interview, and one on the rater composite scores). In the present study, we used the rater composite score from the Italian version of the CAI.^58^

#### Social Cognition.

Social cognition was assessed by the Facial Emotion Identification Test (FEIT)^59^ and The Awareness of Social Inference Test (TASIT).^60^ The FEIT explores emotion perception. It identifies the correct emotion (joy, anger, fear, disgust, surprise, sadness, or neutral) represented in a specific photo.

TASIT is a theory of mind test consisting of 7 scales (positive emotions, negative emotions, simple sarcasm, paradoxical sarcasm, sarcasm enriched, and lie), organized into 3 sections: Emotion recognition using complex information (face expression, gestures, verbal utterances; TASIT1), social inference-minimal (TASIT2), and social inference-enriched (TASIT3). The total scores of FEIT and TASIT were obtained by summing the number of correct answers to each of the individual items.

#### Functional Capacity.

Functional capacity was evaluated using the brief version of the University of California San Diego (UCSD) Performance-based Skills Assessment (UPSA-B).^61^ This performance-based instrument assesses “financial skills” and “communication skills.” A total score ranging from 0 (worst performance) to 100 (best performance) was obtained by summing the 2 domains.

#### Real-Life Functioning.

Real-life functioning was assessed using the Specific Level of Functioning Scale (SLOF),^62,63^ an instrument endorsed by the panel of experts involved in the Validation of Everyday Real-World Outcomes (VALERO) initiative. The SLOF explores several different domains of functioning. The ratings are based on key caregiver’s judgment of the behavior and functioning of patients. The SLOF includes 43 items exploring 6 domains: physical efficiency, skills in self-care, interpersonal relationships, social acceptability, everyday life skills (e.g., shopping, using public transportation), and work skills. In the present study, we only analyzed the 3 SLOF domains showing moderate functional impairment (interpersonal relationships, everyday life skills, and work skills); ceiling effects were observed for the other domains, and there was a reduced variability in patients’ scores. For all SLOF scales, higher scores correspond to better real-life functioning. SLOF scores are attributed by an expert clinician based on the information provided by the caregivers.^63^ To avoid halo effects and overlap between the evaluations carried out with the SLOF and the CAI, different researchers were involved in evaluating real-life functioning and illness-related factors (such as psychopathology or cognitive impairment) in our study.^16^

### Statistical Analyses

**Fig. 1.**
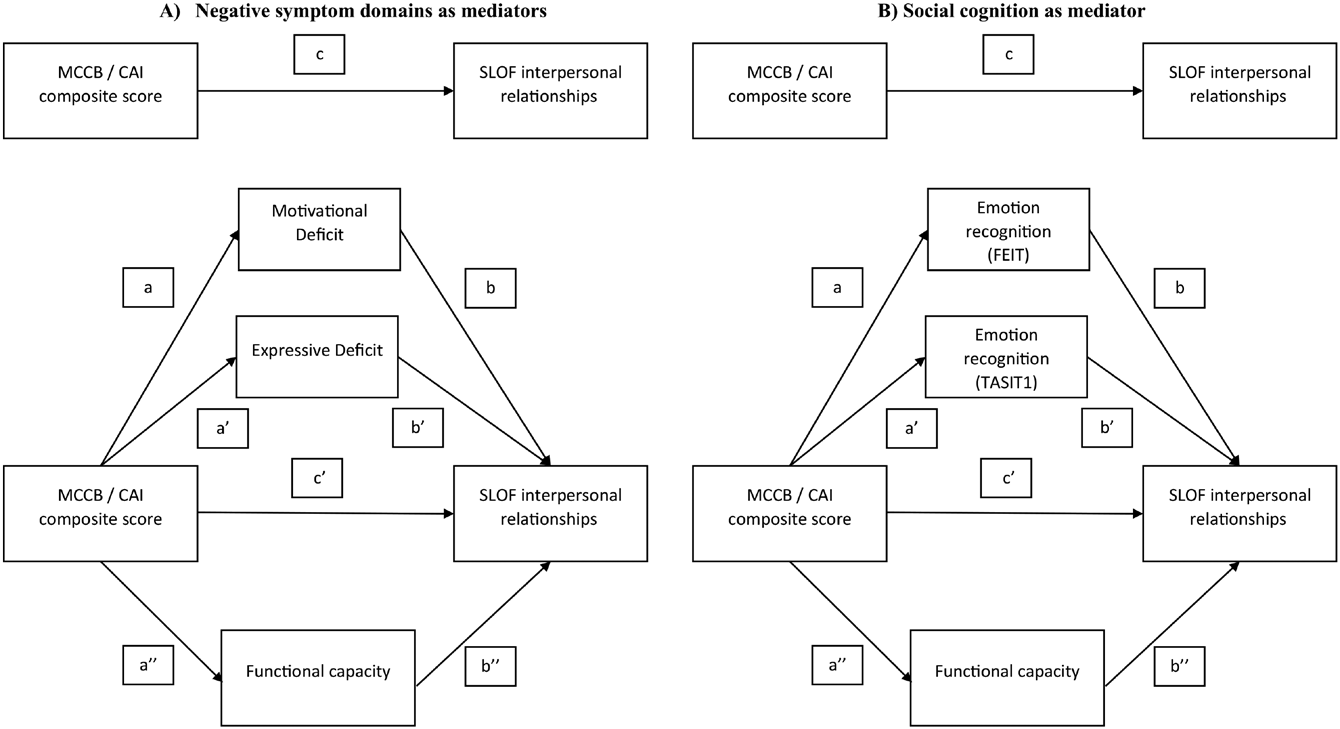
Basic model of mediation analyses with (A) negative symptom domains and (B) social cognition (FEIT and TASIT1) as mediators between neurocognition and SLOF interpersonal relationships. *Note:* MCCB, MATRICS Consensus Cognitive Battery; CAI, Cognitive Assessment Interview; SLOF, Specific Level of Functioning Scale; Int, interpersonal relationships; FEIT, Facial Emotion Identification Test; TASIT, The Awareness of Social Inference Test; MATRICS, Measurement and Treatment Research to Improve Cognition in Schizophrenia.

**Fig. 2.**
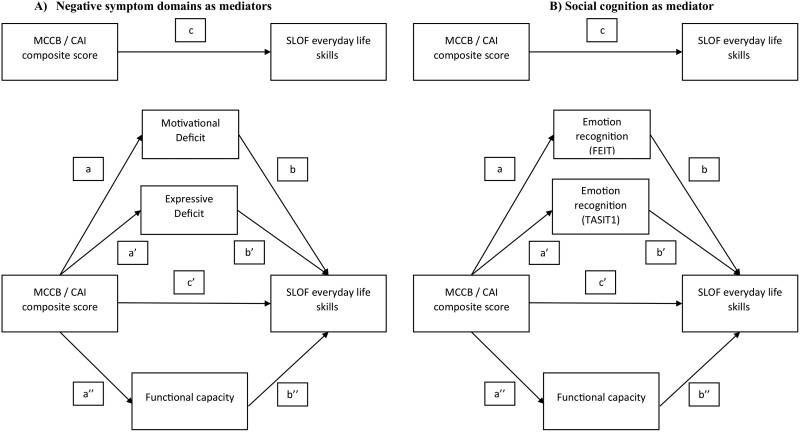
Basic model of mediation analyses with (A) negative symptom domains and functional capacity and (B) social cognition (FEIT and TASIT1) and functional capacity as mediators between neurocognition and SLOF everyday life skills. *Note:* MCCB, MATRICS Consensus Cognitive Battery; CAI, Cognitive Assessment Interview, SLOF, Specific Level of Functioning Scale; Int, interpersonal relationships; FEIT, Facial Emotion Identification Test; TASIT, The Awareness of Social Inference Test; MATRICS, Measurement and Treatment Research to Improve Cognition in Schizophrenia

**Fig. 3.**
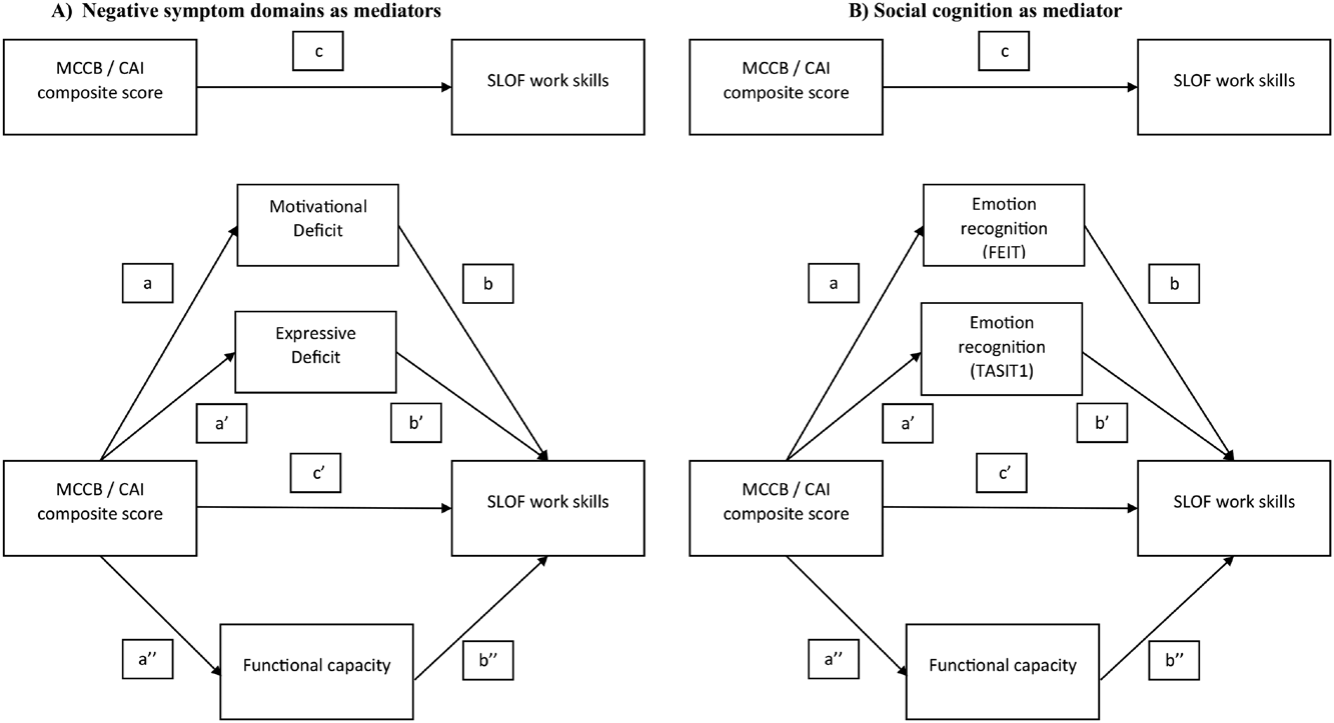
Basic model of mediation analyses with (A) negative symptom domains and (B) social cognition (FEIT and TASIT1) as mediators between neurocognition and measures of functional outcome. *Note:* MCCB, MATRICS Consensus Cognitive Battery; CAI, Cognitive Assessment Interview, SLOF, Specific Level of Functioning Scale; Int, interpersonal relationships; FEIT, Facial Emotion Identification Test; TASIT, The Awareness of Social Inference Test; MATRICS, Measurement and Treatment Research to Improve Cognition in Schizophrenia

Mediation analyses were performed using PROCESS to determine the significance of the indirect effects (mediation) in different models (figures 1, 2, and 3). The same outcome variables, for each model, were investigated: (1) SLOF interpersonal relationships (figure 1), (2) SLOF everyday life skills (figure 2), and (3) SLOF work skills (figure 3). Either the variable “MCCB neurocognition composite score” or the “CAI neurocognition composite score” was the independent variable or predictor, the 2 domains of negative symptoms (motivational deficit and expressive deficit) and functional capacity or social cognition and functional capacity were tested as mediator variables.

To perform the mediation analyses, the predictor has to satisfy the assumption of a linear relationship with the outcome (figures 1–3—path c); the mediator has to demonstrate a linear relationship with both the predictor (figures 1–3—path a/a’/a’’) and the outcome (figures 1–3—path b/b’/b’’). Partial mediation occurs when, after introducing the mediator variable into the model (negative symptom domains and functional capacity in figures 1A, 2A, and 3A; emotion recognition and functional capacity in figures 1B, 2B, and 3B), the direct effect (figures 1–3 – path c’) is reduced compared to the total direct effect (figures 1–3—path c) but is still significant. Complete mediation occurs when the direct effect (figures 1–3—path c’) is no longer significant after introducing the mediator variable.

In our conceptual models (figures 1–3), the effect of neurocognition (MCCB or CAI) on functioning is referred to as the total effect (path c). In the mediation analyses that considered negative symptom domains (Motivational Deficit and Expressive Deficit) and functional capacity as mediators (figures 1A, 2A, and 3A), the total effects included a direct effect of neurocognition on the functional outcome (path c’) and an indirect effect of neurocognition on functioning through the 2 negative symptom domains and functional capacity (mediation paths: a/b, a’/b’, a’’/b’’). In the mediation analyses in which emotion recognition (FEIT and TASIT1) and functional capacity were considered as a mediator (figures 1B, 2B, and 3B), the overall effects included a direct effect from neurocognition to functional outcome (path c’) and an overall indirect effect from neurocognition to functioning via emotion recognition (FEIT and TASIT1) and functional capacity (mediation paths: a/b, a’/b’, a’’/b’’). The mediation analyses are considered valid when the significant level is maintained with 5000 bootstrapping samples and the 95% confidence interval of the indirect effect does not include zero.

All statistical analyses were performed using SPSS software version 25.

## Results

### Subjects

Six hundred and eighteen subjects with a diagnosis of schizophrenia according to the DSM-5 criteria were included in the study. They were 427 men and 191 women, had a mean age of 45.1 ± 10.5 years, and a mean education of 11.7 ± 3.4 years. Demographic and clinical characteristics of the experimental sample are reported in table 1.

**Table 1.** Demographic, Clinical, and Functional Characteristics of the Experimental Sample (*N* = 618)

Variables	Percentage frequency or mean ± SD
Gender (% males)	69.1
Age (years, mean ± SD)	45.1 ± 10.5
Education (years, mean ± SD)	11.7 ± 3.4
PANSS positive factor	8.4 ± 4.3
PANSS disorganization item (P2)	2.4 ± 1.4
BNSS experiential domain	18.6 ± 9.7
BNSS expressive deficit domain	12.0 ± 7.7
MCCB composite score	31.31 ± 12.80
CAI composite score	3.39 ± 1.36
FEIT	37.3 ± 8.1
TASIT 1	20.4 ± 4.8
UPSA-B total score	68.6 ± 23.9
SLOF interpersonal relationships	21.2 ± 6.0
SLOF everyday life skills	46.2 ± 8.3
SLOF work skills	20.1 ± 6.1

*Note:* PANSS, The Positive and Negative Syndrome Scale; BNSS, the Brief Negative Symptom Scale; MCCB, MATRICS Consensus Cognitive Battery; CAI, The Cognitive Assessment Interview; FEIT, Facial Emotion Identification Test; TASIT, The Awareness of Social Inference Test; UPSA-B, the brief version of the University of California San Diego (UCSD) Performance-based Skills Assessment; SLOF, Specific Level of Functioning Scale; MATRICS, Measurement and Treatment Research to Improve Cognition in Schizophrenia.

### Mediation Analysis

The following mediation analyses illustrate the role of negative symptoms or social cognition and functional capacity in mediating the effects of neurocognition (evaluated either using MCCB or CAI) on functional outcomes.

#### Negative Symptoms and Functional Capacity as Mediators Between Neurocognition and Functional Outcome.

The mediation analysis between neurocognition, assessed using MCCB and CAI, are presented in tables 2 and 3, respectively.

**Table 2.** Statistics From the Analyses With Negative Symptoms and Functional Capacity Domains as Mediators Between Neurocognition (MCCB) and Functional Outcome

X	Y	M	*N*	a/a’/a’’, estimates (CI)	b/b’/b’’, estimates (CI)	c, estimates (CI)	c’, estimates (CI)	Indirect effect estimate (CI)
Model 1								
MCCB Composite score	SLOF Int	MAP	511	*−0.252 (−0.31, −0.19)*	*−0.313 (−0.387, −0.240)*	*0.103 (0.062,0.144)*	0.013 (−0.033,0.060)	*0.078 (0.052,0.108)* ^*^
MCCB Composite score	SLOF Int	EXP	511	*−0.238 (−0.28, −0.19)*	.005 (−0.094,0.105)			^†^
MCCB Composite score	SLOF Int	UPSA−B	511	*1.11 (0.98, 1.24)*	.011 (−0.014,0.037)			^†^
Model 2								
MCCB Composite score	SLOF ELS	MAP	509	*−0.252 (−0.31, −0.19)*	*−0.122 (−0.217, −0.026)*	*0.33 (0.072,0.038)*	*0.08 (0.019,0.139)*	*−0.030 (−0.006, −0.057)* ^*^
MCCB Composite score	SLOF ELS	EXP	509	*−0.238 (−0.28, −0.19)*	*−0.194 (−0.322, −0.065)*			*0.046 (−0.012,0.083)* ^*^
MCCB Composite score	SLOF ELS	UPSA-B	509	*1.11 (0.98, 1.24)*	*0.154 (0.121,0.187)*			*0.172 (−0.129,0.218)* ^*^
Model 3								
MCCB Composite score	SLOF WS	MAP	510	*−0.252 (−0.31, −0.19)*	*−0.119 (−0.19, −0.05)*	*−0.193 (0.155,0.230)*	*−0.072 (0.028,0.116)*	*0.030 (0.012,0.052)* ^*^
MCCB Composite score	SLOF WS	EXP	510	*−0.238 (−0.28, −0.19)*	−0.188 (−0.18,0.01)			^†^
MCCB Composite score	SLOF WS	UPSA-B	510	*1.11 (0.98, 1.24)*	*0.062 (0.04,0.09)*			*−0.07 (−0.040, −0.099)* ^*^

*Note:* MCCB, MATRICS Consensus Cognitive Battery; SLOF, Specific Level of Functioning Scale; Int, interpersonal relationships; ELS, everyday life skills; WS, work skills; MAP, Motivational deficit; EXP, Expressive deficit; UPSA-B, University of California San Diego (UCSD) Performance-based Skills Assessment; MATRICS, Measurement and Treatment Research to Improve Cognition in Schizophrenia.

In italics, *P* ≤ .05.

^*^Prerequisites to test for mediation are fulfilled, and the 95% CI of the indirect effect does not include zero and is therefore significant.

^†^Prerequisites to test for mediation are not fulfilled.

The results showed that (1) the direct effect of neurocognition on SLOF interpersonal relationships became nonsignificant, suggesting complete mediation through the motivational deficit domain; (2) the direct effect of neurocognition on SLOF everyday life skills was reduced but remained significant, suggesting partial mediation through both negative symptom domains and functional capacity; (3) the direct effect of neurocognition on SLOF work skills was reduced but remained significant, suggesting partial mediation through both motivational deficit domain and functional capacity.

**Table 3.** Statistics From the Analyses With Negative Symptoms and Functional Capacity as Mediators Between Neurocognition (CAI) and Functional Outcome

X	Y	M	*N*	a/a’/a’’, estimates (CI)	b/b’/b’’, estimates (CI)	c, estimates (CI)	c’, estimates (CI)	Indirect effect estimate (CI)
Model 7								
CAI Composite score	SLOF Int	MAP	566	*−3.29 (−2.75, −3.82)*	*−0.29 (−0.36, −0.22)*	*−1.34 (−1.70, −0.98)*	−0.28 (−0.70,. 41)	*−0.95 (−1.26, −0.65)* ^*^
CAI Composite score	SLOF Int	EXP	566	*−2.87 (−2.46, −3.29)*	.01 (−0.08,0.10)			^†^
CAI Composite score	SLOF Int	UPSA-B	566	*−9.91 (−11.14, −8.67)*	.01 (−0.01,0.04)			^†^
Model 8								
CAI Composite score	SLOF ELS	MAP	564	*−3.28 (−2.74, −3.82)*	−0.08 (−0.17,0.01)	*−3.40 (−3.88, −2.81)*	*−1.06 (−1.60, −0.52)*	^†^
CAI Composite score	SLOF ELS	EXP	564	*−2.87 (−2.45, −3.29)*	*−0.19 (−0.31, −0.07)*			*−0.55 (−0.95, −0.18)* ^*^
CAI Composite score	SLOF ELS	UPSA-B	564	*−9.91 (−11.14, −8.67)*	*0.15 (−0.12,0.18)*			*−1. 51 (−1.88, −1.17)* ^*^
Model 9								
CAI Composite score	SLOF WS	MAP	565	*−3.29 (−2.75, −3.83)*	*−0.08 (−0.14, −0.01)*	*−2.15 (−2.48, −1.82)*	*−1.11 (−1.51, −0.070)*	*−0.25 (−0.50, −0.04)* ^*^
CAI Composite score	SLOF WS	EXP	565	*−2.88 (−2.46, −3.29)*	−0.07 (−0.15,0.02)			^†^
CAI Composite score	SLOF WS	UPSA-B	565	*−9.88 (−11.12, −8.64)*	*.06 (0.04,0.08)*			*−0.60 (−0.81, −0.39)* ^*^

*Note:* CAI, Cognitive Assessment Interview; SLOF, Specific Level of Functioning Scale; Int, interpersonal relationships; ELS, everyday life skills; WS, work skills; MAP, Motivational deficit; EXP, Expressive deficit; UPSA-B, University of California San Diego (UCSD) Performance-based Skills Assessment.

In italics, *P* ≤ .05.

^*^Prerequisites to test for mediation are fulfilled, and the 95% CI of the indirect effect does not include zero and is therefore significant.

^†^Prerequisites to test for mediation are not fulfilled.

The results showed that (1) the direct effect of neurocognition on SLOF interpersonal relationships became nonsignificant, suggesting complete mediation through the motivational deficit domain; (2) the direct effect of neurocognition on SLOF everyday life skills was reduced but remained significant, suggesting partial mediation through expressive deficit domain and functional capacity; (3) the direct effect of neurocognition on SLOF work skills was reduced but remained significant, suggesting partial mediation through both motivational deficit domain and functional capacity.

The total effects of neurocognition on SLOF interpersonal relationships were statistically significant either using MCCB and CAI. Introducing negative symptom domains and functional capacity as mediators, the direct effect became nonsignificant when neurocognition was assessed via MCCB, suggesting complete mediation through the motivational deficit domain (supplementary figure S1 A), while it was reduced but remained significant when assessed through CAI, indicating partial mediation by the same negative symptom domain (supplementary figure S1 B).

The total effect of neurocognition on SLOF everyday life skills was significant both with MCCB and CAI assessments. In both cases, negative symptoms and functional capacity had a partial mediation effect. When neurocognition was evaluated using MCCB, this partial mediation involved negative symptom domains and functional capacity (supplementary figure S3 A). Conversely, with the CAI assessment, the mediation was significant for the expressive deficit domain and functional capacity (supplementary figure S3 B).

Neurocognition had a significant effect on SLOF work skills, regardless of the assessment method and demonstrated a partial mediation effect through both the motivational deficit domain and the functional capacity (supplementary figure S5 A-B).

#### Social Cognition and Functional Capacity as Mediators Between Neurocognition and Functional Outcome.

The mediation analysis between neurocognition, assessed using MCCB and CAI, and functional outcome, with emotion recognition (evaluated with both FEIT and TASIT1) and functional capacity as mediators, yielded significant results, as presented in tables 4 and 5, respectively.

**Table 4.** Statistics From the Analyses With Social Cognition and Functional Capacity Domains as Mediators Between Neurocognition (MCCB) and Functional Outcome

X	Y	M	*N*	a/a’/a’’, estimates (CI)	b/b’/b’’, estimates (CI)	c, estimates (CI)	c’, estimates (CI)	Indirect effect estimate (CI)
Model 4								
MCCB Composite score	SLOF Int	FEIT	469	*0.253 (−0.199, −0.306)*	0.075 (−0.005,0.156)	*0.101 (0.057,0.144)*	−0.025 (−0.32,0.82)	^†^
MCCB Composite score	SLOF Int	TASIT1	469	*−0.201 (−0.172, −0.230)*	*0.158 (0.007,0.309)*			*0.031 (0.003,0.062)* ^*^
MCCB Composite score	SLOF Int	UPSA-B	469	*1.09 (0.96, 1.23)*	.022 (−0.008,0.053)			^†^
Model 5								
MCCB Composite score	SLOF ELS	FEIT	467	*0.253 (−0.199, −0.306)*	0.039 (−0.053,0.131)	*0.101 (0.057,0.144)*	−0.025 (−0.32,0.82)	^†^
MCCB Composite score	SLOF ELS	TASIT1	467	*−0.201 (−0.172, −0.230)*	*0.266 (0.094,0.439)*			*0.053 (0.012,0.098)* ^*^
MCCB Composite score	SLOF ELS	UPSA-B	467	*1.09 (0.96, 1.23)*	*0.158 (0.123,0.193)*			*0.172 (0.126,0.221)* ^*^
Model 6								
MCCB Composite score	SLOF WS	FEIT	468	*0.253 (−0.199, −0.306)*	*0.124 (0.054,0.194)*	*0.199 (0.159,0.239)*	*0.072 (0.023,0.121)*	*0.031 (0.012,0.053)* ^*^
MCCB Composite score	SLOF WS	TASIT1	468	*−0.201 (−0.172, −0.230)*	*0.164 (0.034,0.295)*			*0.033 (0.007,0.061)* ^*^
MCCB Composite score	SLOF WS	UPSA-B	468	*1.09 (0.96, 1.23)*	*0.058 (0.031,0.084)*			*0.063 (0.031,0.094)* ^*^

*Note:* MCCB, MATRICS Consensus Cognitive Battery; SLOF, Specific Level of Functioning Scale; Int, interpersonal relationships; ELS, everyday life skills; WS, work skills; FEIT, Facial Emotion Identification Test; TASIT, The Awareness of Social Inference Test; UPSA-B, University of California San Diego (UCSD) Performance-based Skills Assessment; MATRICS, Measurement and Treatment Research to Improve Cognition in Schizophrenia.

In italics, *P* ≤ .05.

^*^Prerequisites to test for mediation are fulfilled, and the 95% CI of the indirect effect does not include zero and is therefore significant.

^†^Prerequisites to test for mediation are not fulfilled.

The results showed that (1) the direct effect of neurocognition on SLOF interpersonal relationships became nonsignificant, suggesting complete mediation through social emotion recognition (assessed with TASIT1); (2) the direct effect of neurocognition on SLOF everyday life skills became nonsignificant, suggesting complete mediation through social emotion recognition (assessed with TASIT1) and functional capacity; (3) the direct effect of neurocognition on SLOF work skills was reduced but remained significant, suggesting partial mediation through social emotion recognition (assessed with both FEIT and TASIT1) and functional capacity.

**Table 5.** Statistics From the Analyses With Social Cognition and Functional Capacity as Mediators Between Neurocognition (CAI) and Functional Outcome

X	Y	M	*N*	a/a’/a’’, estimates (CI)	b/b’/b’’, estimates (CI)	c, estimates (CI)	c’, estimates (CI)	Indirect effect estimate (CI)
Model 10								
CAI Composite score	SLOF Int	FEIT	483	*−1.93 (−2.47, −1.39)*	*.090 (0.014,0.165)*	*−1.49 (−1.9, −1.1)*	*−1.11 (−1.59, −0.62)*	*−0.17 (−0.34, −0.03)* ^*^
CAI Composite score	SLOF Int	TASIT1	483	*−1.49 (−1.79, −1.19)*	0.121 (−0.021,0.263)			^†^
CAI Composite score	SLOF Int	UPSA-B	483	*−9.81(−11.17, −8.46)*	0.002 (−0.027,0.031)			^†^
Model 11								
CAI Composite score	SLOF ELS	FEIT	481	*−1.94 (−2.47, −1.39)*	0.037 (−0.05,0.12)	*−3.46 (−3.97, −2.95)*	*−1.72 (−2.28, −1.17)*	^†^
CAI Composite score	SLOF ELS	TASIT1	481	*−1.49 (−1.79, −1.19)*	*0.20 (0.037,0.36)*			*−0.30 (−0.63, −0.02)* ^*^
CAI Composite score	SLOF ELS	UPSA-B	481	*−9.81(−11.17, −8.46)*	*0.14 (0.106,0.17)*			*−0.137 (−1.80, −0.097)* ^*^
Model 12								
CAI Composite score	SLOF WS	FEIT	482	*−1.93 (−2.47, −1.39)*	*0.13 (0.06,0.19)*	*−2.34 (−2.70, −1.98)*	*−1.47 (−1.88, −1.06)*	*−0.25 (−0.42, −0.11)* ^*^
CAI Composite score	SLOF WS	TASIT1	482	*−1.49 (−1.79, −1.19)*	*0.13 (0.01,0.25)*			*−0.19 (−0.39, −0.01)* ^*^
CAI Composite score	SLOF WS	UPSA-B	482	*−9.81(−11.17, −8.46)*	*0.04 (0.02, 07)*			*−0.42 (−0.69, −0.18)* ^*^

*Note:* CAI, Cognitive Assessment Interview; SLOF, Specific Level of Functioning Scale; Int, interpersonal relationships; ELS, everyday life skills; WS, work skills; FEIT, Facial Emotion Identification Test; TASIT, The Awareness of Social Inference Test; UPSA-B, University of California San Diego (UCSD) Performance-based Skills Assessment.

In italics, *P* ≤ .05.

^*^Prerequisites to test for mediation are fulfilled, and the 95% CI of the indirect effect does not include zero and is therefore significant.

^†^Prerequisites to test for mediation are not fulfilled.

The results showed that (1) the direct effect of neurocognition on SLOF interpersonal relationships was reduced but remained significant, suggesting partial mediation through social emotion recognition (assessed with FEIT); (2) the direct effect of neurocognition on SLOF everyday life skills was reduced but remained significant, suggesting partial mediation through social emotion recognition (assessed with TASIT1) and functional capacity; (3) the direct effect of neurocognition on SLOF work skills was reduced but remained significant, suggesting partial mediation through social emotion recognition (assessed with both FEIT and TASIT1) and functional capacity.

While the total effect of neurocognition on SLOF interpersonal relationships was significant for both assessments, the type of mediation differed. When assessed via MCCB, the direct effect of neurocognition became nonsignificant, indicating complete mediation by emotion recognition (evaluated by TASIT1; supplementary figure S2 A). Conversely, although the direct effect was reduced with CAI assessment, it remained significant, suggesting a partial mediation effect of emotion recognition (evaluated by FEIT; supplementary figure S2 B).

Likewise, the total effects of neurocognition on SLOF everyday life skills were significant using both assessment methods. However, the nature of the mediation varied between the 2 assessments. While the direct effect became nonsignificant when neurocognition was assessed via MCCB, suggesting complete mediation by emotion recognition (evaluated by TASIT1) and functional capacity (supplementary figure S4 A), the direct effect remained significant, albeit reduced, when assessed through CAI, indicating partial mediation by the same factors (supplementary figure S4 B).

Finally, the significant total effect of neurocognition on SLOF work skills was observed with both assessments. Mediation analysis showed a consistent pattern suggesting a partial mediation effect involving both emotion recognition (evaluated by FEIT and TASIT1) and functional capacity (supplementary figure S6 A-B).

## Discussion

The present study extends the validation of the CAI as a coprimary measure that provides valid information on the impact of cognitive impairment on real-life functioning and on possible mediators of this impact, comparable to those obtained using the MCCB. Indeed, the mediation role of negative symptom domains, functional capacity, and social cognition on the relationships between neurocognition, as assessed using MCCB, and real-life functioning have been confirmed using the CAI to measure neurocognitive functioning.

Our study demonstrated that CAI, in this large sample of community-dwelling subjects with patients with established illness and clinically stable individuals affected by schizophrenia, showed good agreement with performance-based measures like MCCB regarding the relationships with real-life functioning domains. Therefore, the CAI appears to be a reliable coprimary measure, allowing clinicians to capture the impact of cognitive impairment on everyday functioning in individuals with schizophrenia.

In line with the relevant literature, the present study explored the direct and indirect relationships among neurocognition, social cognition, negative symptoms, functional capacity, and real-life functioning in individuals with schizophrenia. The findings provide valuable insight into the mediating roles of negative symptoms (motivational and expressive deficit domains), functional capacity, and social cognition (emotion recognition and theory of mind)on the impact of neurocognition on specific domains of functional outcomes.

Consistently with existing literature,^10,11,21^ the results of our study demonstrated that negative symptoms mediated the relationship between neurocognition, assessed using MCCB, with several areas of functional outcome, confirming their significant role in linking neurocognitive impairments to specific domains of functioning, with the MAP and EXP domains showing different mechanisms. In particular, as regards the MAP domain, our findings demonstrated a full mediation of the impact of neurocognitive deficits on interpersonal relationships and a partial mediation of the neurocognitive impairment impact on everyday life skills and work skills. These findings have been confirmed by the mediation analysis using the CAI, except for everyday life skills. This implies that the presence of motivational deficits could partially explain the influence of neurocognitive deficits on these functional outcomes. The EXP domain, on the other hand, showed a complete mediation effect between neurocognition (assessed either using MCCB or CAI) and everyday life skills. Taken altogether, these findings emphasize the importance of considering specific domains of negative symptoms when examining the impact of neurocognition on functional outcomes and have significant clinical implications, especially regarding the influence of the MAP domain, which has been proven to influence the effectiveness of cognitive remediation in improving interpersonal functioning.^42,64^

In addition to negative symptoms, social emotion recognition, as assessed using FEIT and TASIT1, was found to significantly mediate neurocognition and several areas of functioning. Specifically, our results indicated that impaired emotion recognition completely mediated the relationship between neurocognition (MCCB) and the outcomes of interpersonal relationships and everyday life skills (SLOF). In contrast, emotion recognition partially mediated the relationship between neurocognition and work skills. These findings have been confirmed by the mediation analysis using the CAI.

The mediating role of social cognition has also been addressed in previous studies. Pinkham and colleagues^65^ found that social cognition explained the variability in functional outcomes beyond neurocognition, both in first-episode and chronic schizophrenia, showing promise as a mediator between neurocognition and functional outcomes. Multiple investigations corroborated the role of social cognition as a possible mediator of the impact of neurocognitive impairment on functional outcomes.^42,66–68^ Specifically, 2 previous studies found that emotion recognition mediates neurocognition’s effect on social functioning.^42,69^ This suggests that deficits in identifying and understanding emotions could significantly contribute to social and other aspects of daily functioning impairments in individuals with schizophrenia. This evidence holds significant implications for clinical practice. For instance, the associations identified in this study between social abilities and functioning underscore the importance of including social cognition alongside neurocognition in functional recovery programs. Indeed, meta-analyses indicate that specific interventions focusing on social cognitive remediation can effectively address deficits in social cognition^70,71^

Regarding functional capacity, our mediation analysis confirmed its role as a key mediator between neurocognition, assessed with MCCB, and real-life functioning, specifically with everyday life skills and work skills. These findings were confirmed by the mediation analysis that employed the CAI to measure neurocognition. The results align with the findings reported in previous studies.^11,72–74^ Indeed, a network analysis carried out in a large sample of patients with schizophrenia showed that functional capacity and everyday life skills emerged as the most central and closely linked nodes^11^; in particular, functional capacity served as a bridge, connecting cognition (both neurocognition and social cognition) to everyday life skills. This node, in turn, exhibited connections to other crucial aspects of functioning, such as work skills and interpersonal relationships.^11^

Interestingly, when performing the mediation analysis with the CAI as a measure of neurocognition, the mediation role of negative symptoms, functional capacity, and social cognition was largely confirmed, thereby demonstrating that this instrument might effectively complement the comprehensive performance-based assessment carried out using MCCB and provide similar information on target variables to address to promote recovery. These findings further validate the utility of the CAI as a coprimary measure in clinical trials and support extended use in routine clinical practice when more complex assessment batteries are not available, not only to detect the cognitive impairment of subjects with schizophrenia but also to evaluate the other relevant variables that mediate neurocognitive impact on real-life functioning. CAI effectively provides both clinicians and patients with a clear understanding of cognitive deficits and the specific areas requiring targeted cognitive training interventions. Differently from performance-based scales, which establish a threshold for deficits based on population norms, often understood solely by expert clinicians, the interview-based approach of the CAI might improve patients’ and relatives’ awareness of their cognitive limitations.^26^ The use of the CAI highlights areas where patients struggle to perform real-life tasks, thus emphasizing the importance of training those cognitive domains. Our results highlight that CAI can also be used in mediation analyses to investigate the impact of other areas of impairment, such as negative symptoms, social cognition, or functional capacity, which might limit the generalization of improvement of cognitive functions to real-life tasks. It should be noted that obtaining reliable information from an informant well-acquainted with the patient’s everyday life can be challenging in some situations. This information may not be available for all patients, and the validity of the assessment can be compromised when relying solely on patient self-report. Comparing the scores attributed by expert clinicians relying on either the patients’ or the informants’ reports we found that they were comparable for both the CAI and the SLOF^26,75^ but that several illness-related factors could reduce the patients’ awareness of their functional impairment.

It’s crucial to emphasize that our results demonstrate that the CAI is a valid coprimary measure, allowing us to glean insight into the mediators of cognitive impairment's impact on functioning in people with schizophrenia. The CAI can promote the awareness of how cognition affects daily functioning and what factors might facilitate or reduce such an impact in a manner that is understandable not only for healthcare professionals but also for patients and caregivers, thus complementing the information provided by the formal assessment of cognition using the MCCB. When comparing the effects of neurocognition on functional outcomes using either the MCCB or the CAI, some differences in the mediation results were noteworthy.

For instance, the mediation of motivational deficit in the relationship between neurocognition and everyday life skills is absent when CAI is used, leaving only the EXP domain as a mediator. In other studies, such as the aforementioned network analysis,^11^ there is a strong connection between the EXP domain and functional capacity but not between the MAP domain and everyday life skills. Perhaps, due to the weaker relationship between MAP and everyday life skills, its mediation role is not captured when using the CAI to assess neurocognition.

Additionally, in the case of interpersonal relationships, TASIT, instead of FEIT, acted as a mediator in the assessment conducted through CAI. However, it is important to consider that the composite score of the CAI includes the score of social cognition, which cannot be subtracted. Therefore, it cannot be directly compared to the cognitive score of the MCCB, as it incorporates a component of social cognition.^34^ This likely results in the CAI capturing a different severity of the emotion recognition (as assessed by TASIT 1, which requires integration of facial expression and gestures in social contexts) compared to what the MCCB captures, which seems to be related to more basic emotion recognition skills (recognition of basic emotions in static facial expression). These discrepancies highlight the importance of carefully considering the context and main aims of cognitive assessment in schizophrenia. When the MCCB can be used for a comprehensive assessment of CIAS, the CAI should be used as a coprimary measure; however, when no other formal assessment is available, the CAI represents a useful screening tool, providing valuable insight into the main cognitive problems of the patients and their impact on real-life functioning.

Our results should be interpreted considering some strengths: the substantial sample size comprising individuals with schizophrenia residing within the community; (b) the utilization of state-of-the-art assessment tools for both performance-based and interview-based assessments of neurocognition, in addition to evaluations of psychopathology and real-life functioning. However, it is important to acknowledge several limitations of this study: (1) the absence of the CAI in the baseline study, thereby impeding an exploration of its changes over time; (2) the limited generalizability of findings to patients in their first-episode or acute phases, as the study focused on clinically stable patients with a chronic course of the illness, with minimal positive, disorganization and depressive symptoms; (3) the inclusion of a score of social cognition in the composite score of the CAI, making it not directly comparable to the neurocognitive composite score of the MCCB. These differences may affect the interpretation of results, particularly when comparing the mediation analysis results. As regards social cognition, the interview-based assessment instrument of social cognition, the Observable Social Cognition Rating (OSCARS)^76^ seems to be a promising complement for assessing the impacts of social cognition in future studies. This scale provides a more comprehensive assessment of social cognition than the CAI and could be used alongside it. However, since the OSCARS had not been validated in Italian, it was not used in this study; (4) CAI scores are rater composites, whereas SLOF scores, as validated in the Italian network study,^63^ are attributed by a clinician based on the information provided by the caregivers; therefore, the scores could be partially overlapping. However, to avoid halo effects and overlap between the evaluations carried out with the SLOF and the CAI, different researchers were involved in evaluating real-life functioning and illness-related factors (such as psychopathology or cognitive impairment) in our study. Furthermore, although the information for the SLOF and CAI scores was provided by the same caregiver, the interviews are completely different and investigate different areas of functional impairments. Indeed, our results demonstrate that although they are associated, these associations are not as high as they could be if they were to explore the same domains of functional impairment.

In conclusion, the CAI is a highly recommendable coprimary measure for assessing cognition in schizophrenia alongside performance-based assessment instruments. Our extensive multicenter study showed good agreement between CAI and performance-based measures and showcased CAI efficacy in assessing deficits in neurocognitive domains and the impact of the same domains on real-life functioning in individuals with schizophrenia.

These findings suggest the potential utility of the CAI in clinical trials. It could serve as a complementary measure alongside neuropsychological tests, offering assessments that are more relevant to patients and caregivers than raw neuropsychological scores.

Moreover, the CAI could find application in routine clinical practice as a swift and convenient screening tool for overall cognitive impairment in patients. It would provide valuable insights into patients’ perceptions of how their cognitive deficits affect their functional outcomes and potentially boost motivation to participate in cognitive rehabilitation programs aimed at improving functional outcomes.^77,78^

Additionally, adding interview-based tools, like the CAI, to performance-based assessments could be essential for achieving a comprehensive understanding of schizophrenia complexity and its impact on various cognitive and functional domains for clinicians, patients, and caregivers.

## Supplementary Material

sgae020_suppl_Supplementary_Materials

## Data Availability

The original contributions presented in the study are included in the article, further inquiries can be directed to the corresponding author/s.
