# Supplementary material for: Interview Versus Performance Assessment of Cognition as Predictors of Real-World Outcomes in a Large-Scale Cross-Sectional Study in Schizophrenia
Source: Schizophr Bull Open. 2024 Aug 12;5(1):sgae020. doi: 10.1093/schizbullopen/sgae020 (PMC11362673; doi:10.1093/schizbullopen/sgae020)
Supplement: sgae020_suppl_Supplementary_Materials [file sgae020_suppl_supplementary_materials.docx]

**Interview versus performance-based assessment of cognition as predictors of real-world outcomes in a large-scale cross-sectional study in subjects with schizophrenia.**

**Index**

**Figure S1. Mediation analyses with negative symptoms and functional capacity as mediators between neurocognition, assessed either using the MATRICS Consensus Cognitive Battery (MCCB) (A) or the Cognitive Assessment Interview (CAI) (B) and the real-life functional outcome “Interpersonal relationships” as assessed using the Specific Level of Functioning Scale (SLOF).**

**Figure S2. Mediation analyses with social cognition evaluated with both the Facial Emotion Identification Test (FEIT) and The Awareness of Social Inference Test (TASIT) and functional capacity as mediators between neurocognition, assessed either using the MATRICS Consensus Cognitive Battery (MCCB (A) or the Cognitive Assessment Interview (CAI) (B) and the real-life functional outcome “Interpersonal relationships” as assessed using the Specific Level of Functioning Scale (SLOF).**

**Figure S3. Mediation analyses with negative symptoms and functional capacity as mediators between neurocognition, assessed either using the MATRICS Consensus Cognitive Battery (MCCB) (A) or the Cognitive Assessment Interview (CAI) (B) and the real-life functional outcome “Everyday life skills” as assessed using the Specific Level of Functioning Scale (SLOF).**

**Figure S4. Mediation analyses with social cognition evaluated with both the Facial Emotion Identification Test (FEIT) and The Awareness of Social Inference Test (TASIT) and functional capacity as mediators between neurocognition, assessed either using the MATRICS Consensus Cognitive Battery (MCCB (A) or the Cognitive Assessment Interview (CAI) (B) and the real-life functional outcome “Everyday life skills” as assessed using the Specific Level of Functioning Scale (SLOF).**

**Figure S5. Mediation analyses with negative symptoms and functional capacity as mediators between neurocognition, assessed either using the MATRICS Consensus Cognitive Battery (MCCB) (A) or the Cognitive Assessment Interview (CAI) (B) and the real-life functional outcome “Work skills” as assessed using the Specific Level of Functioning Scale (SLOF).**

**Figure S6. Mediation analyses with social cognition evaluated with both the Facial Emotion Identification Test (FEIT) and The Awareness of Social Inference Test (TASIT) and functional capacity as mediators between neurocognition, assessed either using the MATRICS Consensus Cognitive Battery (MCCB (A) or the Cognitive Assessment Interview (CAI) (B) and the real-life functional outcome “Work skills” as assessed using the Specific Level of Functioning Scale (SLOF).**

**Figure S1. Mediation analyses with negative symptoms and functional capacity as mediators between neurocognition, assessed either using the MATRICS Consensus Cognitive Battery (MCCB) (A) or the Cognitive Assessment Interview (CAI) (B) and the real-life functional outcome “Interpersonal relationships” as assessed using the Specific Level of Functioning Scale (SLOF).**

**The motivational deficit domain is a full mediator of the impact of neurocognition on Interpersonal relationships (A) when using MCCB to assess cognition. In contrast, the motivational deficit domain partially mediates the impact of neurocognition on Interpersonal relationships (B) when CAI is used to assess cognition.**

**A B**

MCCB

SLOF Interpersonal Relationships

UPSA-B

MAP

EXP

CAI

SLOF Interpersonal Relationships

UPSA-B

MAP

EXP

**MCCB: MATRICS Consensus Cognitive Battery; MAP: Motivational domain; EXP: Expressive deficit domain; UPSA-B: Brief UCSD Performance-based Skills Assessment; CAI: Cognitive Assessment Interview; SLOF: Specific Level of Functioning Scale.**

**A solid arrow indicates a significant relationship between variables. The arrow's thickness indicates the strength of the relationship: thicker arrows represent stronger relationships. A dashed arrow indicates a non-significant relationship between variables.**

**Figure S2. Mediation analyses with social cognition (evaluated with both the Facial Emotion Identification Test (FEIT) and The Awareness of Social Inference Test (TASIT)) and functional capacity as mediators between neurocognition, assessed either using the MATRICS Consensus Cognitive Battery (MCCB (A) or the Cognitive Assessment Interview (CAI) (B) and the real-life functional outcome “Interpersonal relationships” as assessed using the Specific Level of Functioning Scale (SLOF).**

**Emotion recognition (evaluated by TASIT1) is a full mediator of the impact of neurocognition on Interpersonal relationships (A) when using MCCB to assess cognition. In contrast, emotion recognition (evaluated by FEIT) partially mediates the impact of neurocognition on Interpersonal relationships (B) when CAI is used to assess cognition.**

MCCB

SLOF Interpersonal Relationships

UPSA-B

FEIT

TASIT1

CAI

UPSA-B

FEIT

TASIT1

**A B**

SLOF Interpersonal Relationships

.

**MCCB: MATRICS Consensus Cognitive Battery; FEIT: Facial Emotion Identification Test; TASIT1: The Awareness of Social Inference Test – section 1; UPSA-B: Brief UCSD Performance-based Skills Assessment; CAI: Cognitive Assessment Interview; SLOF: Specific Level of Functioning Scale.**

**A solid arrow indicates a significant relationship between variables. The arrow's thickness indicates the strength of the relationship: thicker arrows represent stronger relationships. A dashed arrow indicates a non-significant relationship between variables.**

**Figure S3. Mediation analyses with negative symptoms and functional capacity as mediators between neurocognition, assessed either using the MATRICS Consensus Cognitive Battery (MCCB) (A) or the Cognitive Assessment Interview (CAI) (B) and the real-life functional outcome “Everyday life skills” as assessed using the Specific Level of Functioning Scale (SLOF).**

**Both negative symptom domains and functional capacity are partial mediators of the impact of neurocognition on Everyday life skills (A) when using MCCB to assess cognition. In contrast, only the expressive deficit domain partially mediates the impact of neurocognition on Everyday life skills (B) when CAI is used to assess cognition.**

**A B**

MCCB

SLOF

Everyday Life Skills

UPSA-B

MAP

EXP

CAI

SLOF

Everyday Life Skills

UPSA-B

MAP

EXP

**MCCB: MATRICS Consensus Cognitive Battery; MAP: Motivational domain; EXP: Expressive deficit domain; UPSA-B: Brief UCSD Performance-based Skills Assessment; CAI: Cognitive Assessment Interview; SLOF: Specific Level of Functioning Scale.**

**A solid arrow indicates a significant relationship between variables. The arrow's thickness indicates the strength of the relationship: thicker arrows represent stronger relationships. A dashed arrow indicates a non-significant relationship between variables.**

**Figure S4. Mediation analyses with social cognition (evaluated with both the Facial Emotion Identification Test (FEIT) and The Awareness of Social Inference Test (TASIT)) and functional capacity as mediators between neurocognition, assessed either using the MATRICS Consensus Cognitive Battery (MCCB (A) or the Cognitive Assessment Interview (CAI) (B) and the real-life functional outcome “Everyday life skills” as assessed using the Specific Level of Functioning Scale (SLOF).**

**Emotion recognition (evaluated by TASIT1) and functional capacity are full mediators of the impact of neurocognition on Everyday life skills (A) when using MCCB to assess cognition. In contrast, the same variables partially mediate the impact of neurocognition on Everyday life skills (B) when CAI is used to assess cognition.**

**A B**

MCCB

SLOF

Everyday Life Skills

UPSA-B

FEIT

TASIT1

CAI

SLOF

Everyday Life Skills

UPSA-B

FEIT

TASIT1

**MCCB: MATRICS Consensus Cognitive Battery; FEIT: Facial Emotion Identification Test; TASIT1: The Awareness of Social Inference Test – section 1; UPSA-B: Brief UCSD Performance-based Skills Assessment; CAI: Cognitive Assessment Interview; SLOF: Specific Level of Functioning Scale; ELS = everyday life skills.**

**A solid arrow indicates a significant relationship between variables. The arrow's thickness indicates the strength of the relationship: thicker arrows represent stronger relationships. A dashed arrow indicates a non-significant relationship between variables.**

**Figure S5. Mediation analyses with negative symptoms and functional capacity as mediators between neurocognition, assessed either using the MATRICS Consensus Cognitive Battery (MCCB) (A) or the Cognitive Assessment Interview (CAI) (B) and the real-life functional outcome “Work skills” as assessed using the Specific Level of Functioning Scale (SLOF).**

**The motivational deficit domain and functional capacity are partial mediators of the impact of neurocognition on Work skills when using both (A) MCCB and (B) CAI to assess cognition.**

**A B**

MCCB

SLOF

Work Skills

UPSA-B

MAP

EXP

CAI

SLOF

Work Skills

UPSA-B

MAP

EXP

**MCCB: MATRICS Consensus Cognitive Battery; MAP: Motivational domain; EXP: Expressive deficit domain; UPSA-B: Brief UCSD Performance-based Skills Assessment; CAI: Cognitive Assessment Interview; SLOF: Specific Level of Functioning Scale; WS = work skills.**

**A solid arrow indicates a significant relationship between variables. The arrow's thickness indicates the strength of the relationship: thicker arrows represent stronger relationships. A dashed arrow indicates a non-significant relationship between variables.**

**Figure S6. Mediation analyses with social cognition (evaluated with both the Facial Emotion Identification Test (FEIT) and The Awareness of Social Inference Test (TASIT)) and functional capacity as mediators between neurocognition, assessed either using the MATRICS Consensus Cognitive Battery (MCCB (A) or the Cognitive Assessment Interview (CAI) (B) and the real-life functional outcome “Work skills” as assessed using the Specific Level of Functioning Scale (SLOF).**

**Both emotion recognition (evaluated by FEIT and TASIT1) and functional capacity are partial mediators of the impact of neurocognition on Work skills when using both (A) MCCB and (B) CAI to assess cognition.**

MCCB

SLOF

Work Skills

UPSA-B

FEIT

TASIT1

CAI

SLOF

Work Skills

UPSA-B

FEIT

TASIT1

**A B**

**MCCB: MATRICS Consensus Cognitive Battery; FEIT: Facial Emotion Identification Test; TASIT1: The Awareness of Social Inference Test – section 1; UPSA-B: Brief UCSD Performance-based Skills Assessment; CAI: Cognitive Assessment Interview; SLOF: Specific Level of Functioning Scale; WS: work skills.**

**A solid arrow indicates a significant relationship between variables. The arrow's thickness indicates the strength of the relationship: thicker arrows represent stronger relationships. A dashed arrow indicates a non-significant relationship between variables.**
